# The Impact of Asymptomatic Helminth Co-Infection in Patients with Newly Diagnosed Tuberculosis in North-West Ethiopia

**DOI:** 10.1371/journal.pone.0042901

**Published:** 2012-08-29

**Authors:** Ebba Abate, Meseret Belayneh, Aschalew Gelaw, Jonna Idh, Assefa Getachew, Shitaye Alemu, Ermias Diro, Nigussu Fikre, Sven Britton, Daniel Elias, Abraham Aseffa, Olle Stendahl, Thomas Schön

**Affiliations:** 1 Department of Immunology and Molecular Biology, University of Gondar, Gondar, Ethiopia; 2 Department of Medical Microbiology, Linköping University, Linköping, Sweden; 3 Medical Faculty, School of Medical Laboratory Sciences, Addis Ababa University, Addis Ababa, Ethiopia; 4 Department of Radiology, University of Gondar, Gondar, Ethiopia; 5 Department of Internal Medicine, University of Gondar, Gondar, Ethiopia; 6 Department of Parasitology, Medical Faculty, Addis Ababa University, Addis Ababa, Ethiopia; 7 Department of Infectious Diseases, Karolinska Hospital, Stockholm, Sweden; 8 University of Southern Denmark, Institute of Molecular Medicine, Department of Cancer and Inflammation, Odense, Denmark; 9 Armauer Hansen Research Institute, Addis Ababa, Ethiopia; 10 Department of Clinical Microbiology, Kalmar County Hospital, Kalmar, Sweden; University of Iowa Carver College of Medicine, United States of America

## Abstract

**Background:**

Areas endemic of helminth infection, tuberculosis (TB) and HIV are to a large extent overlapping. The aim of this study was to assess the impact of asymptomatic helminth infection on the immunological response among TB patients with and without HIV, their house hold contacts and community controls.

**Methodology:**

Consecutive smear positive TB patients (n = 112), their household contacts (n = 71) and community controls (n = 112) were recruited in Gondar town, Ethiopia. Stool microscopy, HIV serology, serum IgE level, eosinophil and CD4 counts were performed and tuberculosis patients were followed up for 3 months after initiation of anti-TB treatment.

**Results:**

Helminth co-infection rate was 29% in TB patients and 21% in both community control and household contacts (p = 0.3) where *Ascaris lumbricoides* was the most prevalent parasite. In TB patients the seroprevalence of HIV was 47% (53/112). Eosinophilia and elevated IgE level were significantly associated with asymptomatic helminth infection. During TB treatment, the worm infection rate of HIV+/TB patients declined from 31% (10/32) at week 0 to 9% (3/32) at week 2 of TB treatment, whereas HIV−/TB patients showed no change from baseline to week 2, 29% (13/45) vs. 22.2% (10/45). This trend was stable at week 8 and 12 as well.

**Conclusion:**

One third of smear positive TB patients were infected with helminths. Eosinophilia and elevated IgE level correlated with asymptomatic worm infection, indicating an effect on host immunity. The rate of worm infection declined during TB treatment in HIV+/TB co-infected patients whereas no decline was seen in HIV−/TB group.

## Introduction

Infections caused by intestinal helminths are associated to divergent animal groups and are highly prevalent worldwide, affecting populations living in poor regions [1]. Intestinal helminths are reported to induce a Th-2 type immunity in the host [2] and evidence suggests that the Th2 immune response may play a crucial role in reducing the severity of acute disease upon helminth infection [3]. The immune response of the host to worm infection correlates with the production of interleukin 4 (IL-4), IL -5, IL-9, IL-10, and IL-13 and consequently the development of strong Immunoglobulin E (IgE) and eosinophilia [3].

Studies have indicated that the humoral immune response to parasites favours infection by *Mycobacterium tuberculosis* and that helminth infected individuals could be at risk for developing tuberculosis (TB) [4]. A case control study conducted in Ethiopia has shown that the prevalence of worms in active TB patients was higher than in their healthy household contacts [5]. This observation is supported by other studies conducted in different areas [4], [6].

The association between eosinophilia and protective immunity in human subjects came from the post-treatment, re-infection studies in schistosomiasis that demonstrated a direct relationship between the lack of re-infection and the level of peripheral blood eosinophils [7]–[8]. It was furthermore shown that up-regulation of Th2 responses including eosinophilia and IgE hyper responsiveness [9] by helminthic infection can suppress the production of a Th1 immune response which is important to combat intracellular pathogens such as *M. Tuberculosis* [5], [10]–[11]. Previous reports have also indicated that regulatory T cells are expanded in patients with TB and may contribute to suppression of Th1-type immune responses [12]. In this study, we assessed the magnitude of worm infection in TB patients in Gondar town compared to household contacts and community controls and investigated the relation of surrogate markers to Th2 type immune response at TB diagnosis, and the rate of helminth infection during follow up after TB treatment in relation to levels in household contacts and community controls.

## Materials and Methods

### Patients

Consecutive newly diagnosed smear positive tuberculosis patients between the ages of 15–65 years were recruited at the Directly Observed Treatment Short-course (DOTS) clinic at University of Gondar Hospital (GUH) and Gondar Health Centre, Ethiopia. Smear positive TB was defined as at least two sputum smears positive for acid fast bacilli (AFB) or one smear positive slide and x-ray results suggestive of TB. All individuals, irrespective of age, who lived together with TB patients in the same house and had no previous TB history or any evidence of TB on chest x-ray were recruited as house hold contacts (HC). Apparently healthy blood donors who visited the blood bank at the GUH during the study period, and had passed pre-donation clinical screening to rule out any chronic illnesses and previous TB history, were enrolled as community controls (CC). Hospitalized patients, pregnant women and those with concomitant disease other than HIV were excluded from participation. TB patients were followed up after enrolment at weeks 2, 8 and 12. None of the study participants had symptoms suggestive of helminth infection during enrolment or follow up. As per the HIV treatment programme in Ethiopia, HIV positive patients were put on anti- retroviral treatment and/or cotrimoxazole prophylactic treatment based on the working national guideline taking the WHO HIV clinical stage and CD4 count into account.

### HIV serology and CD4 count

HIV screening was determined from whole blood samples using rapid HIV kits, Determine [(Determine® HIV-1/2 Ag/Ab Combo, US), Capillus (Trinity Biotech, US) and Unigold (Trinity Biotech, US)]. As per the national HIV testing algorithm, samples negative by Determine were reported as negative. But if the test became positive, the second test, Capillus, was done. The HIV result was then reported according to the results of Capillus test. i.e If the result of Capillus became concordant with Determine (positive test), the result was reported as positive. But for any discordant result of Determine and Capillus, the third test, Unigold, was used as tie breaker and the final result was decided based on the results obtained from Unigold test.

CD4 count was performed using FacsScan machine (BD, USA) in 4 ml venous blood samples according to the manufactures' instructions.

### Assessment of helminth infection and surrogate markers for Th2 type immune response

Three stool samples were collected using a strategy where one sample was collected daily for three consecutive days from each participant and then examined both using direct and Kato-Katz technique [13] by the same technician throughout the study. The results of positive or negative were based on the examination of all three samples together from each patient. One in 10 slides were randomly selected and checked again blindly by a second microscopist for quality control. Serum IgE was determined with a commercial ELISA kit (Immundiagnostik, Germany) according to the manufacturer's instruction. The absolute eosinophil count of peripheral blood was computed in mm^3^ from the value of total and differential white blood cell counts obtained using Cell Dyn 1800 (Abbot, USA).

### Ethical statement

Written informed consent was obtained from all study participants. Written consent was obtained from the respective guardians for children below the age of 18. The study was approved by the Ethical Review Board of the University of Gondar, Ethiopia and by the Regional Ethics Review Board, Linköping, Sweden.

### Data analysis

Data are presented in tables as median and inter quartile range. A p-value of <0.05 was considered as statistically significant. Significance testing was done with Mann Whitney and Wilcoxon tests for continuous data and Fisher's exact test for discrete variables. Variables with a p<0.1 in the univariate analysis were entered into a multiple regression analysis with helminth status as the dependent variable. The regression analysis was performed using the Statistica software (Tusla, USA). For the remaining statistical analysis Epinfo 2005 (version 3.5.1) was used.

## Results

### Baseline clinical data

A total of 295 study participants were included in the study. Of these, 112 were TB patients, 112 community controls (CC) and the remaining 71 were household contacts (HC) with median age of 28, 30 and 20 respectively (Table 1). A significantly higher HIV co-infection rate was identified among TB patients (47%) compared to CC (1.8%, p<0.001) and HC (11.6%, p<0.01). Within the TB group women were more commonly co-infected with HIV than men (58% vs 36%, p = 0.016). Intestinal helminths were identified from 29% (32/112) of the TB patients, 21% (23/112) of the community controls and 21% (15/71) of the house hold contacts. There was no statistical difference in the prevalence of helminths between TB patients and a combined or separate analysis of the CC and HC groups (p = 0.16). *Ascaris lumbricoides* was the most common intestinal parasite observed in all three groups followed by *Hook worm* (Table 2). The median CD4 count of HIV negative TB patients was 513 cells/mm^3^ and this was significantly lower than the median CD4 count of HIV negative HC (714 cells/mm^3^; p = 0.012, Table 3).

**Table 1:** Comparison of immunological characteristics among participants in TB patients, community controls and house hold contacts.

Variables	TB (n = 112)	CC (n = 112)	HC (n = 71)	p-value
Gender (Median age) : M	53 (28)	101(30)	27(17)	
F	59(27)	11(25)	44(25)	
Median HIV positive; n (%)	53 (47%)	2 (1.8%)	8/69 (11.6%)	<0.001*; <0.01**
Median CD4 in HIV negative (iqr)	513 (390–682)	750(627–923)	714 (578–825)	0.32*; 0.012**
Median eosinophil of helminth positive (iqr)	234 (116–424)	600 (492–960)	602 (270–850)	<0.001*;0.005**
Median IgE of Helminth positive (iqr)	351 (145.5–913)	378 (188–507)	420 (127–760)	<0.001*; <0.001**

Significant differences between groups are depicted with their respective p-values.

* TB patients compared with CCs (community contros);

** TB patients compare with HCs (household contacts).

**Table 2:** Intestinal helminths identified among study participants included in TB patients, community controls and house hold contacts.

*Type of parasites examined*	Groups		
	TB (n = 32)	CCs (n = 23)	HCs (n = 15)
	n (%)	n (%)	n (%)
*Ascaris lumbricoides*	12 (38)	14 (61)	5 (33)
*Hook worm*	8 (25)	12 (52)	5 (33)
*Shistosoma mansoni*	6 (19)	3 (13)	3 (20)
*Trichurs trichuira*	8 (25)	1 (4)	1 (7)
*Strongyloide stercoralis*	3 (9)	0	0
*Hymenolopis nana*	0	1 (4)	0
*Taenia spps.*	0	0	1 (7)

**Table 3:** Immunological characteristics with respect to helminth status among participants in the TB, community control and household contact groups.

	TB (n = 112)			CCs (n = 112)			HHCs (n = 71)		
	Helminth positive	Helminth negative	p-value	Helminth positive	Helminth negative	p-value	Helminth positive	Helminth negative	p-value
	n = 32	n = 80		n = 23	n = 89		n = 15	n = 56	
HIV positive, (%)	13 (25)	19 (68)	0.25	1 (4)	1 (1)	0.74	1 (7)	7 (13)	0.44
CD4, median (iqr)	310 (120–602)	314 (144–518)	0.58	650 (592–757)	781 (645–949)	0.21	653 (605–752)	714 (554–855)	0.5
Eosinophilia,	46% (10/22)	20% (10/50)	0.028*	94% (17/18)	65% (34/52)	0.02*	69% (9/13)	48% (10/21)	0.38
>300 cells/mm^3^									
Eosinophilia,	23% (5/22)	8% (4/50)	0.05	72% (13/18)	52% (27/52)	0.07	62% (8/13)	33% (7/21)	0.06
>500 cells/mm^3^									
Elevated IgE,	75% (18/24)	51% (33/65)	0.033*	94% (17/18)	62% (32/52)	0.01*	77% (10/13)	14% (3/21)	0.0008*
>120 IU/l									

### Impact of asymptomatic co-infection on eosinophilia and IgE levels

Eosinophilia (>300 cells/mm^3^) was correlated with helminth infection in TB group (p = 0.028) and community controls (p = 0.02). Similarly, elevated IgE (>120 IU/L) correlated with helminth infection in TB patients (p = 0.033), community controls (p = 0.01) and house hold contacts (p = 0.0008), (Table 3). In a multivariate regression analysis in the TB patients, eosinophilia (>500 cells/mm3; adjusted OR: 15.2; 95% CI: 1.4–160.3, p = 0.02) and increased IgE-levels (>120 kU/L, adjusted OR: 7.6; 95% CI: 1.2–48.4. p = 0.03) were independently associated with asymptomatic helminth infection, which was not confounded by sex or HIV-serostatus. TB patients co-infected with helminths had lower median eosinophil counts compared to community controls (234 cells/mm^3^ vs 600 cells/mm^3^, p<0.001) and household contacts (234 cells/mm^3^ vs. 602 cells/mm^3^, p = 0.005) (Table 1). Similarly, helminth co-infected TB patients had lower median IgE levels at base line compared to community controls (351 IU/L vs. 378 IU/L, p<0.001) and house hold contacts (351 IU/L vs. 420 IU/L, p<0.001) infected with helminths (Table 1).

### Reduction in the rate of helminth infection in HIV coinfected patients following initiation of treatment against tuberculosis

**Figure 1:**
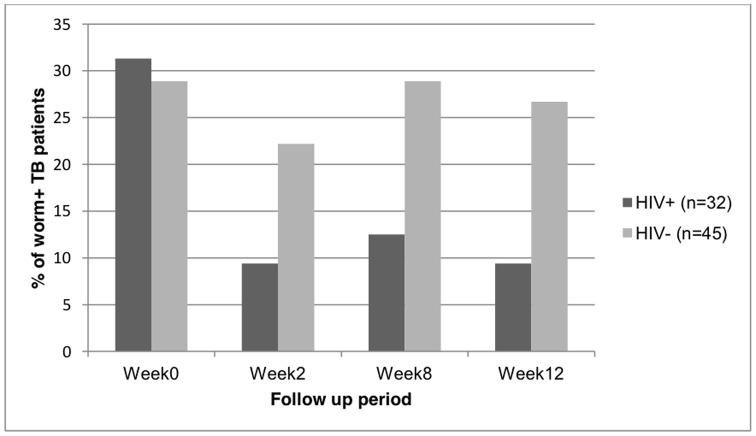
Trends of helminth status of HIV positive and HIV negative patients during TB treatment.

We observed a rapid decline in worm burden of HIV+/TB patients after 2, 8 and 12 weeks of TB treatment. The worm infection rate of HIV+/TB patients declined rapidly from 31% (10/32) at week 0 to 9% (3/32) at week 2, whereas HIV−/TB patients showed no change, with 29% (13/45) at week 0 and 22% (10/45) at week 2. This trend was stable at week 8 and after 3 months (Figure 1). Of the total 77 TB patients for whom we had all follow up stool results, 86% [91% (29/32) of HIV positive and 82% (37/45) of HIV negative] had the same positive or negative worm status throughout the follow up (at week 2, week 8 and month 3) as at week 0.

## Discussion

Of the total 295 study participants included in the three groups, 24% were positive for intestinal parasites without any clinical symptoms suggestive of active infection. The co-infection rate of helminths among TB patients in the present study is similar with a report from Brazil [14]. Although still controversial, some studies suggested that the humoral immune response to parasites favours infection by *Mycobacterium tuberculosis* and thus the immune response to intestinal parasites could be a risk factor for developing tuberculosis [4]. This has been supported in studies conducted in different areas showing high association of helminth infection among TB patients, however the causality could not be definitely confirmed [4], [6]. In this study, we present further support for the hypothesis that even asymptomatic helminth infection affect the immune system as both eosinophilia (>300 cells/mm^3^) and increased IgE levels (120 KU/L) were found in the helminth infected groups of TB patients, community controls and house hold contacts. The TB co-infection rate with intestinal parasites in this study (29%) was less than the 72% and 41% rates reported in the same hospital in 2006 and 2007 respectively [15]–[16]. Difference in patient selection is a probable explanation for the observed variation since only smear positive TB patients with symptoms suggestive to intestinal helminths were recruited in the study by Elias et al (Daniel Elias, personal communication). In addition, the two studies were conducted during the pre-HIV treatment era and then the start of anti- retroviral therapy (ART) and better care for HIV and TB/HIV patients might have changed the epidemiology.

More than 30 thousands rural health extension workers were trained and currently have been working at community level all over Ethiopia [17]. The major task of this large pool is implementing different preventive health packages including development of latrines and improving hygienic practice at family level which may have direct impact in reducing helminth infection. Thus, the contribution of increasing awareness and improving hygienic practice could have had an impact on an actual decrease in the magnitude of intestinal parasites in the area. *Ascaris lumbricoides* was the prevailing intestinal parasite species identified in the three categories with varying proportions (range from 33% to 61%). In contrast to earlier studies conducted in the area, we found a higher hook worm rate (range from 25% to 52%) observed in the three groups.

Results from human and animal models have shown that eosinophilia and elevated IgE are one effector mechanism of immunity during parasite invasion [18]. Setting of cut off value for eosinophilia using the adopted reference range may not be appropriate for Ethiopian population since previous reports indicated that the hematological reference range of healthy Ethiopians was lower than the adopted reference range [19]. Thus, we used two cut off values to determine eosinophilia (>300 cell/mm3, applicable to Ethiopian population and >500 cells/mm3 which is the commonly used cut off). Accordingly, using cut off value of >300 cells/mm3, eosinophilia was associated with helminth status in the TB group (p = 0.028) and community controls (p = 0.02) but this statistical association was lost at the cut off value of >500 cells/mm3 (p = 0.05 and p = 0.07, respectively). However, increased IgE level was associated with worm infection in TB patients (p = 0.033), community controls (p = 0.01) and house hold contacts (p = 0.008). Thus, our study confirms the hypothesis that asymptomatic helminth infection correlates to effects on host immunity illustrated by increased eosinophil counts and IgE levels.

To our knowledge, no previous reports have followed helminth infection status during anti-TB treatment. There was no on-going routine screening for helminth infection in the area and all patients were asymptomatic and had not received anti-helminthic treatment during the 12 week follow up period. During the study period, participants were closely followed. We observed a rapid and marked decline in worm burden of HIV+/TB patients followed at 2, 8 and 12 weeks after anti-TB treatment. This was supported by the observation that among the 77 TB patients included in the analysis, most of the helminth positive patients, 86% (66/77) had same worm status in accordance to week 0 stool results throughout the follow up at week 2, week 8 and month 3.

Even though the HAART (Highly Active Anti-Retroviral Therapy) -induced reconstitution of cellular immunity would be the main factor for reducing opportunistic infections among HIV patients, *in vitro* and *in vivo* investigations indicated that HIV treatment, especially with protease inhibitors (PIs), could have a direct effect in killing of parasites including malaria [20]–[21]. In addition, an anti-helminthic effect of cotrimoxazole (used as prophylaxis in HIV positives) cannot be excluded, similar to its described anti-malarial effect [22]. Thus, the antiretroviral therapy and effect of cotrimoxazole might have contributed to the rapid decline in worm rate seen in our study without direct anti-helminthic therapy. This requires further studies including more subjects and information on exact ART regimen and co-trimoxazole prophylaxis and the lack of such information is a clear limitation of our study which was not design to explore this difference.

In summary, a high burden of intestinal parasites was observed among TB patient and asymptomatic helminth infection correlated to increased eosinophil count and serum IgE levels indicating an effect on host immunity. Additionally, we noted a rapid decline in the rate of worm infection among HIV+/TB co-infected patients, without direct anti-helminthic treatment, after 2 weeks of TB treatment.
